# Supplementary material for: Complexome profiling of the Chlamydomonas psb28 mutant reveals TEF5 as an early PSII assembly factor
Source: Plant Cell. 2025 Mar 17;37(6):koaf055. doi: 10.1093/plcell/koaf055 (PMC12164590; doi:10.1093/plcell/koaf055)
Supplement: koaf055_Supplementary_Data [file koaf055_supplementary_data.zip › psb28_interactive.zip!/plots/intensity/qualityPlots/peptides/_Cre19.g750247.t1.1.html]

### Cre19.g750247.t1.1

Trivial name:   
SeqCoverage [median]: 16.60  
Mass: 37.85 kDa  
  
 

| Sequence | MissedCleavage | Mass [Da] | wtIntensity | muIntensity | Score |
| --- | --- | --- | --- | --- | --- |
| AAQQDEAPPAPAPTLVGEDSAAFDLAK | 0 | 2679.30 | 644.63 | 1284.25 | 71.08 |
| GEELVGPR | 0 | 855.45 | 5142.65 | 16595.65 | 129.39 |
| LHLWETGIMR | 0 | 1254.65 | 1786.01 | 8041.69 | 66.44 |
| TSVVPFAALADGR | 0 | 1302.69 | 16441.19 | 6344.47 | 131.82 |
|  |  |  | 24014.5 | 32266.1 |  |
